# Early life environment affects behavior, welfare, gut microbiome composition, and diversity in broiler chickens

**DOI:** 10.3389/fvets.2022.977359

**Published:** 2022-09-12

**Authors:** Ingrid C. de Jong, Dirkjan Schokker, Henk Gunnink, Maudia van Wijhe, Johanna M. J. Rebel

**Affiliations:** ^1^Wageningen Livestock Research, Wageningen University and Research, Wageningen, Netherlands; ^2^Wageningen Bioveterinary Research, Wageningen University and Research, Lelystad, Netherlands

**Keywords:** broiler, welfare, microbiome, environment, behavior

## Abstract

This study aimed to identify whether early-life conditions in broiler chickens could affect their behavior and welfare, and whether or not this was associated with an altered gut microbiome composition or diversity. Broilers were tested in a 2 x 2 factorial design with hatching conditions [home pen (OH) or at the hatchery (HH)] and enrichment (dark brooder (EE) or no brooder (NE) until 14 days of age) as factors (*N* = 6 per treatment combination). Microbiota composition was measured in the jejunum on days (d) 7, 14, and 35 and in pooled fecal samples on day 14. A novel environment test (NET) was performed on days 1 and 11, and the behavior was observed on days 6, 13, and 33. On day 35, composite asymmetry was determined and footpad dermatitis and hock burn were scored. In their home pen, HH showed more locomotion than OH (*P* = 0.05), and NE were sitting more and showed more comfort behavior than EE at all ages (*P* <0.001 and *P* = 0.001, respectively). On days 6 and 13 NE showed more eating and litter pecking while sitting, but on day 33 the opposite was found (age^*^enrichment: *P* = 0.05 and *P* <0.01, respectively). On days 1 and 11, HH showed more social reinstatement in the NET than OH, and EE showed more social reinstatement than NE (*P* <0.05). Composite asymmetry scores were lower for EE than NE (*P* <0.05). EE also had less footpad dermatitis and hock burn than NE (*P* <0.001). Within OH, NE had a more diverse fecal and jejunal microbiome compared to EE on day 14 (feces: observed richness: *P* = 0.052; jejunum: observed richness and Shannon: *P* <0.05); the principal component analysis (PCA) showed differences between NE and EE within both HH and OH in fecal samples on day 14, as well as significant differences in bacterial genera such as *Lactobacillus* and *Lachnospiraceae* (*P* <0.05). On day 35, PCA in jejunal samples only showed a trend (*P* = 0.068) for differences between NE vs. EE within the OH. In conclusion, these results suggest that especially the dark brooder affected the behavior and had a positive effect on welfare as well as affected the composition and diversity of the microbiome. Whether or not the behavior was modulated by the microbiome or vice versa remains to be investigated.

## Introduction

An important moment in early life is the rapid microbial colonization of the gut. In chickens, immediately after hatch, the microbiome is established, and a succession of different bacterial species occurs (1–4). Studies have shown that different factors can influence the microbiome composition in the intestine, such as diet (5, 6), genetics (2), geographical location (7), housing system (8), and early life environmental conditions (9). The gut microbiome affects the development and maturation of the host immune system (10, 11), and accumulating evidence suggests that gut microbiota can influence host behavior in rodents and pigs (12–15). Also, in poultry, recent studies identified a relationship between gut microbiome composition and behavior (16–20). A more diverse gut microbiome has been associated with reduced stress levels and better welfare in chickens (21, 22), as well as the presence or relative abundance of certain phyla or genera (21–23). Early-life housing and management can have a profound impact on the chicken's behavioral and immunological development (24–31), and an association with gut microbiome composition has been suggested (23). Thus, there might be an association between the early rearing environment, gut microbiome composition, and behavioral characteristics in poultry, which could influence a bird's ability to cope with environmental and social challenges in production systems (i.e., resilience).

Management in the early life of broiler chickens affects behavior and welfare. In commercial practice, broiler chickens often hatch in the sterile environment of a hatchery and are subsequently transported to the farm. It can take up to 72 h before chickens have access to feed (32), and although they might acquire microorganisms from the environment at the hatchery or during transport (33), this delay in access to feed may affect the microbiota development. Early access to feed is important as there is a dramatic increase in microorganisms in the chicken's intestine after the first ingestion of feed (4), which stimulates the development of the gut and the immune system (34–36). A delay in access to feed affects the development of the gut and the immune system in broilers (26, 35, 37) and gut microbiota composition in laying hens (24). Interestingly, an effect of the delay in access to feed on fearfulness has been found, but only in combination with transportation (38). Alternatively, a place where chickens can access feed and water immediately after hatch, such as an on-farm hatching system, is increasingly being used (39). On-farm hatching can result in better performance, reduced first week or total mortality, and reduced footpad dermatitis (39–43), and it may also affect fear-related responses (42, 44), although the latter could not be confirmed by (45). On-farm hatching may thus have long-term consequences for welfare, health, and production. Yet, it is unknown whether or not these effects might be associated with changes in the gut microbiome.

Also, the physical environment affects the behavior and welfare of chickens. For example, providing access to perches and the ability to engage in natural behaviors decreased heterophil-to-lymphocyte ratios in hens, which is an indicator of chronic stress in chickens (46). Rearing laying hens in an environment with multiple enrichments improved humoral immune response (31), reduced fearful behaviors, and reduced baseline comb temperature and plasma corticosterone concentration, which can also be considered indicators of stress in chickens (23, 47), as compared to rearing in a barren environment. Providing environmental enrichments to broiler chickens stimulates natural behavior (48) and may reduce fear (49, 50), resulting in a more positive affective state determined with a judgment bias test as compared to rearing in a barren environment (51). Moreover, Yan et al. (23) observed an altered cecal microbiome composition and diversity in laying hens housed with complex enrichment compared to hens in a barren environment, and suggested a role of the gut microbiome in the development of behavior, brain, and immune response in layers housed in different environments, although this needs further investigation.

An early-life housing condition that has been shown to have long-term effects on the welfare of laying hens is the environmental enrichment in the form of a dark brooder (52–54). The dark brooder gives chicks access to a warm, secluded, dark area, and a choice between being in an active or inactive group (52). Layer chicks with access to a dark brooder showed reduced fearfulness (53) and less feather pecking (52, 54), a damaging behavior in laying hens that is strongly related to fearfulness and stress (55). However, access to dark brooders did not influence fearfulness or explorative behavior in slow-growing broilers (56) and did not affect the corticosterone level in the feathers of adult laying hens (57). Thus, it remains to be further studied whether a dark brooder indeed affects broiler welfare and whether this might be related to changes in the gut microbiome composition.

The present research aimed to study whether early-life conditions in broiler chickens like on-farm hatching and access to a dark brooder, affect home-pen behavior and fearfulness, other indicators of welfare [contact dermatitis, which is a frequently measured indicator of welfare in broiler chickens (58), and composite asymmetry, reflecting the ability to cope with stressors during the rearing period (59)], gut microbiome composition and diversity, and to identify possible associations between these factors. We hypothesized that on-farm hatching would result in better welfare [less footpad dermatitis (39–43) and lower composite asymmetry score] and reduced fearfulness (42, 44), but would have only minor effects on home-pen behavior (44, 45). Regarding the dark brooder, we hypothesized that this would have a more long-term positive effect on home-pen behavior (more active and comfortable behavior at older ages), as has been observed in laying hens (52–54), reduce fearfulness (53), and also result in a lower composite asymmetry score due to a better ability to cope with stressors. In addition, we expected that both the hatching environment and the dark brooder would affect gut microbiome diversity and composition by affecting early colonization of the gut (24) and increasing environmental complexity (23), respectively.

## Materials and methods

### Ethical approval

The animal study was reviewed and approved by the Ethical Committee of Wageningen University and Research (License Number AVD401002016578).

### Animals and on-farm hatching procedure

The experiment included a total of 576 Ross 308 broiler chickens divided over 24 pens with 24 chickens (12 male, 12 female) on day 0. Two randomly chosen chickens per pen (one male and one female) were euthanized and dissected on days 7, 14, and 35 for the collection of jejunal content (see below). These two chickens received a color mark (one pair until day 7, one pair until day 14, and one pair until day 33), and these two marked chickens were also subjected to the novel environment test on days 1 and 11 (see below).

All chickens in the experiment, both that hatched on-farm and from the hatchery (see below), were from the same batch of eggs and the same parent stock (42 weeks of age). On day 18 of incubation, after candling, egg trays were alternately assigned to one of both treatments in a commercial hatchery (Probroed, The Netherlands). A total of 384 18-day-incubated eggs were transported to the experimental farm of Wageningen University and Research (Lelystad, The Netherlands) and distributed over cardboard trays specially made for on-farm hatching (One2Born, Uden, The Netherlands). Per pen, 32 eggs were placed in one tray (three rows of 10 eggs with an empty row in between, and two additional eggs placed in the middle). After 21 days of incubation, all hatched chickens were sexed and healthy, first-grade chickens were selected for the experiment. This day was named “d0” according to commercial practice. The remaining chickens that were on-farm hatched were removed from the pen and euthanized. On day 0, 384 additional chickens from the same parent stock batch arrived from the hatchery and were randomly placed in the other pens. All female chickens received a wing mark upon placement in the pen or selection on day 0.

### Experimental design, housing, and management

A 2 x 2 factorial design was used, with either on-farm hatched (OH) or hatchery-hatched (HH) chickens and either no enrichment (NE) or a dark brooder as enrichment (EE) until day 14 of age. Treatments were divided into two identical rooms that could be accessed through one common hallway, which was connected to the central hallway of the experimental building. Climate and light settings were identical for both rooms, apart from room temperature until day 14 (see below). In each room, pens were placed in two rows of six on the left and right sides of the room, and these could be accessed from a central corridor in the middle of the room. For the dark brooder treatment, the environmental temperature was reduced compared to the room without the dark brooders, so that the dark brooder was the primary heat source (60). The EE treatment was randomly allocated to one of both rooms. Within each room, pens were alternately assigned to the OH or HH treatment.

Pens were 20 cm apart to prevent contamination between them through the exchange of litter or feces. In addition, on three sides, pens were closed off to 20 cm height from the floor whereas the other side was closed by the feeding troughs (1 m height). Furthermore, to prevent contamination, clean plastic overshoes and gloves were used when caretakers or observers needed to enter a pen and/or catch a chicken. Each pen measured 1.5 x 1 x 1 m (l x w x h). Two food troughs of 75 cm in length each were placed in front of the pen and four drinking nipples with cups were available at the back side of the pen. Fresh wood shavings were used as litter material and these were also present in the OH pens on day 18 of incubation (upon placement of the eggs). In each pen, food was provided on paper and water in small dishes to enable newly hatched chickens to find food and water; this was continued until day 3 when all chickens had started to eat from the trough. In the OH pens, the eggshell temperature was measured two times per day after placement of the eggs until the majority started to hatch to ensure an egg temperature of 37.8°C. On day 0, the cardboard tray and the egg shells were removed from the OH pens.

In the OH pens, the dark brooders were switched on before hatching, and in the HH pens before the placement of day-old chickens to ensure a proper brooder temperature, and the brooder was available to the chickens immediately post-hatch (OH) or upon placement (HH). In addition, in the room with the EE treatment, the environmental temperature was reduced by 2°C below the normal temperature schedule from day 0 to day 14 of age to stimulate the chickens to use the brooder as a warm resting place (52). The dark brooder measured 0.5 x 0.5 m and was accessible *via* black plastic flaps on each side. The height of the brooders was adjusted to the height of the birds two times per week. On day 14, the dark brooders were removed from the pens, and from day 14 onward an equal temperature schedule was applied for both experimental rooms. In the room without the dark brooders, the temperature was set at 35°C on day 0 and decreased to 18°C from day 33 onward. From the placement of the eggs until day 1, 24 h light was provided. From days 1 to 4 18L:6D schedule was applied, and thereafter a 16L:8D schedule. Lights were on from 06:00 h onward. Light intensity was 20 lux at bird height. A standard three-phase pelleted commercial diet (ABZ, Nijkerk, The Netherlands) provided *ad libitum* with a starter from days 0–15, grower from days 15–28, and finisher from day 28 onward. All pens were vaccinated against IB and NCD by spray vaccination at d0.

### Observations

#### Performance

Body weights were determined on days 0, 1, 15, 28, and 35 at pen level. Mortality was recorded daily, with reason (if known). Chickens selected for the dissections were individually weighed upon removal from the pen.

#### Behavior

##### Home pen behavior

Cameras were mounted above the pens to record the behavior of the chickens. Three ages were selected for scan sampling of home pen behavior, i.e., days 6, 13, and 33. Scan sampling was done for 2 h per day during the light period: 08:00–09:00 h and 16:00–17:00 h, and these periods excluded the checking of the birds by the caretakers. For each pen, every 10 min the number of chickens performing one of the behaviors as listed in the ethogram (Table 1) was scored. This resulted in 12 scans per pen per age.

**Table 1:** Ethogram defining the different behavioral categories.

**Behavior**	**Definition**
Eating	Pecking at the feeder/head in feeder
Drinking	Pecking at the nipple or drip cup
Standing	Standing without doing anything else
Locomotion	Walking, running (may be accompanied by wing flapping), flying, jumping (not being part of aggression)
Sitting	Sitting, lying, without doing anything else
Sitting while litter pecking	Sitting while pecking at the litter
Foraging	Standing and pecking and/or scratching the litter
Comfort behavior	Preening, wing flapping, and stretching
Dustbathing	All elements of dustbathing according to Van Liere (61)
Aggression	All elements of aggression such as hopping, threatening, kicking, and aggressive pecking toward other chickens
Not visible/in the brooder	Behavior not visible, or chicken not visible (e.g., because being in the dark brooder for the EE groups)
Other	All other behaviors, including explorative pecking at pen parts, feather pecking, freeze, standing alert, etc. that are not included in the other categories

#### Novel environment test

A novel environment test according to de Haas et al. (25) was carried out by one observer on days 1 and 11 of age for one male and one female per pen; different chickens were tested on days 1 and 11. Test birds received a small color mark on their head after testing for dissections on days 7 and 14, respectively. Both chickens in a pen were tested before moving to the next pen, and all pens in a room were tested before moving to the next room. The test was performed in the small hallway to which the rooms were connected. In short, a chicken was caught from a pen and placed in a black round bucket (23.5 cm in diameter at the bottom and 23.5 cm in height) that served as a novel environment. For 2 min the latency to vocalize, latency to the first escape attempt, number of vocalizations, and the number of escape attempts were scored by the observer that was out of sight of the chicken. Thereafter the bucket was cleaned, and the chicken was placed back in the pen. After testing two chickens, before moving to the next pen, the bucket was thoroughly cleaned with alcohol to prevent contamination between pens.

#### Contact dermatitis and relative and composite asymmetry

On day 35, all broilers per pen were scored for footpad lesions (FPD) and hock burn (HB) according to Welfare Quality (58). Briefly, both FPD and HB were scored on a scale from 0–4, with 0 being no lesion and 4 being a large and deep lesion.

On day 35, four broilers were randomly selected (two males and two females) and euthanized, and the legs were collected by cutting the tibia, a few cm above the metatarsus. Legs were marked for each broiler and frozen at −20°C until further analysis. Before analysis, legs were thawed and the length of the left and right middle toe, and the length and width of the left and right metatarsus were measured using X-ray. Relative metatarsus length was calculated:


$$\begin{array}{l} \;relative\;metatarsus\;length\\ =\;\frac{\left|metatarsus\;length\;left-metatarsus\;length\;right\right|}{(metatarsus\;length\;left+metatarsus\;length\;right)/2} \end{array}$$

This was done in a similar way for relative metatarsus width and middle toe length. From these values, the composite asymmetry score was calculated by the sum of the 3 relative asymmetry scores, as described by (59).

#### Microbiota sampling and analysis

At 14 days of age, feces were collected by cloacal swabbing of six broilers per pen (three males and three females; these were not the marked broilers). Swabs were pooled per pen in a bag and stored at −80°C until further analysis. At 7, 14, and 35 days of age, jejunal content was collected from two broilers per pen (one male and one female) after dissection. The two randomly selected and color-marked broilers per pen (see 2.2) were individually weighed, anesthetized with Zoletil® 100, and killed by cervical dislocation. Samples were frozen on dry ice and stored at −80°C until analysis. To isolate DNA, samples were mixed in a 1:1 ratio with PBS and centrifuged for 5 min at 4°C at 300*xg*. The supernatant was collected and centrifuged for 10 min at 4°C at 9,000*xg*. DNA was extracted from the pellet using the “QIAamp DNA stool minikit” (Qiagen, Valencia, CA, USA) according to manufacturers' instructions, after mechanical shearing of the bacteria in Lysing Matrix B tubes (MP Biomedicals, Solon, OH, USA) using the FastPrep-24 three times for 30 s at a speed of 30 Hz (MP Biomedicals, Solon, OH, USA). Quality and quantity of DNA were checked using the NANOdrop (ND1000, Agilent Technologies, Santa Clara, CA, USA). PCR was used to amplify (20 cycles) the 16S rRNA gene V3 fragment using forward primer V3_F (CCTACGGGAGGCAGCAG) and reverse primer V3_R (ATTACCGCGGCTGCTGG) (62). PCR efficiency was checked on an agarose gel. Amplicons were sequenced using paired-end sequencing, 2x150bp technology on a MiSeq sequencer (Illumina, San Diego, CA, USA) at a sequencing depth in the range of 43, 592–597, 448 read-pairs per sample (median 216, 170 read-pairs per sample) for the total dataset. One sample that did not pass the quality control was already excluded.

For the pooled samples (*n* = 24,) we filtered samples having more than 5,000 merged reads, resulting in 22 samples (excluding one OH-NE and one HH-NE sample) that were subsequently rarefied to an even depth to 27,855, and 1,061 taxa remained for further down-stream analysis.

For the individual samples (*n* = 141), we filtered samples having more than 5,000 merged reads, resulting in 135 samples that were subsequently rarefied to an even depth of 5,215 and 1,762 taxa remained for further down-stream analysis.

### Statistical analysis

Analyses of body weight, behavior, and welfare indicators were performed using GenStat (version 17, VSN International). The normality of the data was checked with residual plots. Measures of body weight were analyzed using a mixed (REML) model with repeated measures to test for the fixed effects of treatment, age, and their interaction. Body weight data were log-transformed before testing. For home pen behavior, the proportion of chickens showing dustbathing was added to the category “comfort behavior” (Table 1), and the proportion of chickens showing aggression could not be analyzed as too many zeros were present in these data. Scan sampling data of the behavior were summed for all scans per behavioral category, per age, and divided by the total number of observed broilers in that session. Data were analyzed by GLMM with a binomial distribution and logit link, with hatching system, enrichment, age, and their interactions as fixed effects, and pen and observer as random effects. Latency to vocalize in the NET was log-transformed before analysis. Latency to vocalize and frequency of vocalizations in the NET were analyzed with ANOVA with treatment (hatching system and enrichment), age, and sex and their interactions as fixed factors and pen^*^age as block. FPD and HB scores were analyzed as ordinal variables with a generalized linear model using a logit link.

For statistical analysis of the fecal and jejunal microbiota diversity and composition, the vegan (v2.5–6) and phyloseq (v1.28.0) packages within the R environment (R version 3.6.1) were used. For the diversity measures, i.e., Observed richness and Shannon index, we used Student's *t*-test to test for the significance of enrichment (EE vs. NE) within the different hatching systems (HH and OH). For ordination, we used a principal coordinate analysis with the Bray–Curtis dissimilarity. To test for statistical significance, first, the seed (an integer vector containing the random number generator (RNG) state for random number generation) was set to “12,345,” and thereafter *adonis* (tests if the position of the centroids differs among the treatments) and *betadisper* (tests if the communities differ in their variance) were performed. To investigate the differences between groups on the phyla and genera levels, stacked-bar plots were generated. Differences of *P* <0.05 were considered statistically significant, 0.05 ≤ P ≤ 0.10 were considered a trend.

## Results

### Performance

Nineteen chickens died or were culled during the experiment, of which five, 3, 5, and 6 chickens for HH-NE, OH-NE, HH-EE, and OH–EE treatments, respectively. For body weight, a significant age^*^hatching system (Wald statistic = 128.3; *P* <0.001) and age^*^enrichment interaction (Wald statistic = 10.93; *P* = 0.018) were found. On day 0, OH chickens were heavier than HH chickens, but this difference disappeared thereafter. On day 1, no treatment differences were observed, whereas, on day 12, HH-NE chickens were most heavy, followed by OH-NE, HH-EE, and OH-EE (Table 2). Thus, chickens with the dark brooder had the lowest body weights on day 12. On day 35, treatments did not differ any more in body weight (Table 2).

**Table 2:** Average body weights (grams) on days 0, 1, 12 and 35 of age.

**Age**	**HH-NE**	**HH-EE**	**OH-NE**	**OH-EE**	**se**
Day 0	42.6^a^	41.8^a^	47.6^b^	47.1^b^	0.3
Day 1	50.8	49.5	51.0	50.2	0.6
Day 12	410.3^c^	391.7^b^	395.5^b^	376.5^a^	4.7
Day 35	2,609	2,595	2,555	2,565	31

Data are presented as back-transformed means ± pooled se. HH, hatchery hatched; OH, on-farm hatched; NE, no brooder; EE, dark brooder until day 14 of age.

^a, b, c^Superscripts within a row lacking a common letter differ significantly (P <0.05).

### Home pen behavior

Table 3 shows the back-transformed means and *P*-values for the behavioral categories where no significant interaction with age was found and Table 4 for significant hatching system^*^age and enrichment^*^age interactions. Hatching system^*^enrichment interactions and three-way interactions were non-significant. The hatching system only had a significant effect on the proportion of chickens showing locomotion; HH chickens showed more locomotion than OH chickens (predicted means (on the logit scale) for locomotion: HH: −3.11, OH: −3.30; se: 0.15; *P* = 0.05) (Table 3). NE chickens showed more comfort behavior as compared to EE chickens (predicted means: NE: −2.1, EE: −2.3; se:0.07; *P* = 0.009) (Table 3). Effects of age were found for drinking, locomotion, foraging, comfort behavior, and others, with, in general, a decrease in the number of chickens being active with age, and an increase in the number of chickens showing comfort behaviors with age (Table 3; predicted means not shown).

**Table 3:** Proportion of chickens performing the different behaviors (back-transformed means) and *P*-values for hatching conditions (HH, hatchery-hatched; OH, on-farm hatched), enrichment (NE, no brooder; EE, dark brooder until 14 days of age), and age. Significant effects are indicated in bold.

	**Hatching system**		**Enrichment**		**Age**				
**Behavior^1^**	**HH**	**OH**	**NE**	**EE**	**6**	**13**	**33**	**P_hatching_**	**P_enrichment_**	**P_age_**
Eat	5.02	4.91						0.74	–	–
Drink	7.56	8.84	7.94	8.04	9.29^a^	9.04^a^	6.05^b^	0.89	0.11	**<0.001**
Stand	2.52	2.78	2.80	2.49	2.76	2.62	2.55	0.33	0.44	0.88
Locomotion	4.26^a^	3.56^b^	3.58	4.23	5.60^a^	4.22^b^	2.48^c^	**0.05**	0.83	**<0.001**
Sit	50.77	50.29						0.65	–	–
Sit -peck^2^	8.43	8.89						0.51	–	–
Forage	1.02	0.14	0.15	1.00	2.09^a^	2.71^a^	0.01^b^	0.31	0.38	**<0.001**
Comfort^3^	10.25	9.71	10.91^a^	9.11^b^	8.92	9.61	11.55	0.53	**0.01**	0.20
Other	2.06	1.78	2.01	1.82	2.03^b^	2.76^a^	1.25^c^	0.89	0.63	**0.003**

An empty cell indicates a significant age*enrichment interaction; these values and corresponding P-values are presented in in this table.

^a, b, c^Superscripts within a row lacking a common letter differ significantly; ^1^Aggression is excluded from analysis, as data included too many zeros; category brooder/not visible is only included in in this table; ^2^Sit-peck: sitting while pecking at the litter; ^3^ Comfort behavior is the sum of dustbathing and other comfort behaviors. Significant effects are indicated in bold.

**Table 4:** Average percentage of chickens eating, sitting, sitting while pecking at the litter and in the brooder/not visible, where a significant interaction between hatching system and age (upper part), and/or enrichment and age (lower part), was found.

**Behavior**	**6-HH**	**6-OH**	**13-HH**	**13-OH**	**33-HH**	**33-OH**	P_hatching*age_
Not visible^1^	5.36^a^	6.21a	2.63^b^	1.48^c^	0^d^	0^d^	0.008
	**6-NE**	**6-EE**	**13-NE**	**13-EE**	**33-NE**	**33-EE**	**P**_enrichment*age_
Eating	5.75^a^	4.00^ac^	4.01^c^	3.88^c^	5.63^b^	7.39^a^	0.05
Sitting	48.25^d^	43.11^f^	52.75^c^	38.26^d^	63.18^a^	57.55^b^	0.005
Sit-peck^2^	9.29^abcd^	8.80^dc^	10.67^a^	8.75^bcd^	6.11^e^	8.42^d^	0.006
Brooder/not visible^1^	2.61c	12.26^b^	0.22^d^	15.68^a^	0^e^	0^e^	<0.001

HH, hatchery-hatched; OH, on-farm hatched; NE, no brooder; EE, dark brooder until 14 days of age. ^a, b, c, d, e, f^Superscripts within a row lacking a common letter differ significantly; ^1^Chickens that could not be identified on the videos, either because of being in a corner of the pen, and/or because these were in the dark brooder (EE treatment); ^2^Sitting while pecking at the litter.

A significant hatching system^*^age interaction was found for the category “not visible” as this was only observed on days 6 and 13, but not on day 33 (Table 4; predicted means not shown). Regarding the EE treatment, a significant age^*^enrichment interaction was found for eating, sitting, and sitting while pecking the litter and not visible/in the brooder. More NE chickens were eating on day 6 and 13, while on day 33, more EE chickens were eating. Predicted means were: day 6, NE: −2.80; d6, EE: −3.18; d13, NE: −3.17, d13, EE: −3.21; d33, NE: −2.53; d33, EE: −2.82; se: 0.20; *P* = 0.05). In addition, NE chickens were sitting more than EE chickens at all ages (predicted means: d6, NE: −0.07; d6, EE: −0.27; d13, NE: 10.99, d13, EE: −0.47; D33, NE: 0.54, D33, EE: 0.30, se: 0.09; *P* = 0.005), whereas the proportion of chickens pecking at the litter while sitting was more or less constant with age for EE, it was higher on days 6 and 13 for NE compared to EE, but lower on day 33 (predicted means day 6, NE: −2.28; day 6, EE: −2.34; day 13, NE: −2.11, day 13, EE: −2.35; day 33, NE: −2.73; day 33, EE: −2.38; se: 0.14; *P* = 0.006). Finally, a significant age^*^treatment interaction was found for the proportion of chickens not visible/in the brooder because the brooder was not present on day 33 (Table 4; predicted means not shown). Back transformed indicates that the brooder was well-used on days 6 and 13 (Table 4), even if we assume that a few percent of these young chicks were not visible on the videos at all because of obstruction or they were too small, as was observed in the NE groups.

#### Novel environment test

**Figure 1:**
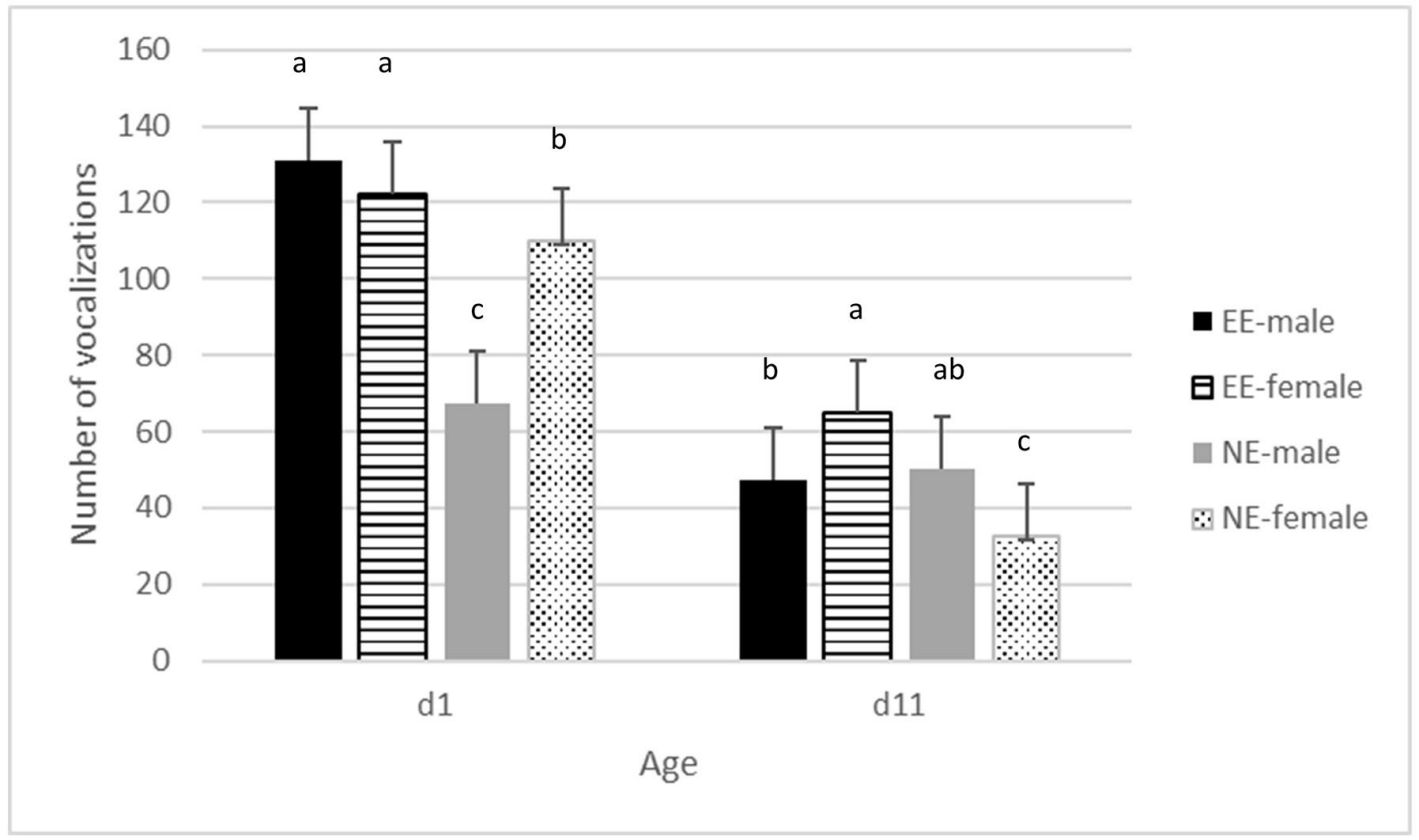
Average frequency of vocalizations ± se in the novel environment test on days 1 and 11 of age, for males and females in pens without a brooder (NE) and with a dark brooder until day 14 of age (EE). Bars lacking a common letter differ significantly (*P* <0.05).

**Figure 2:**
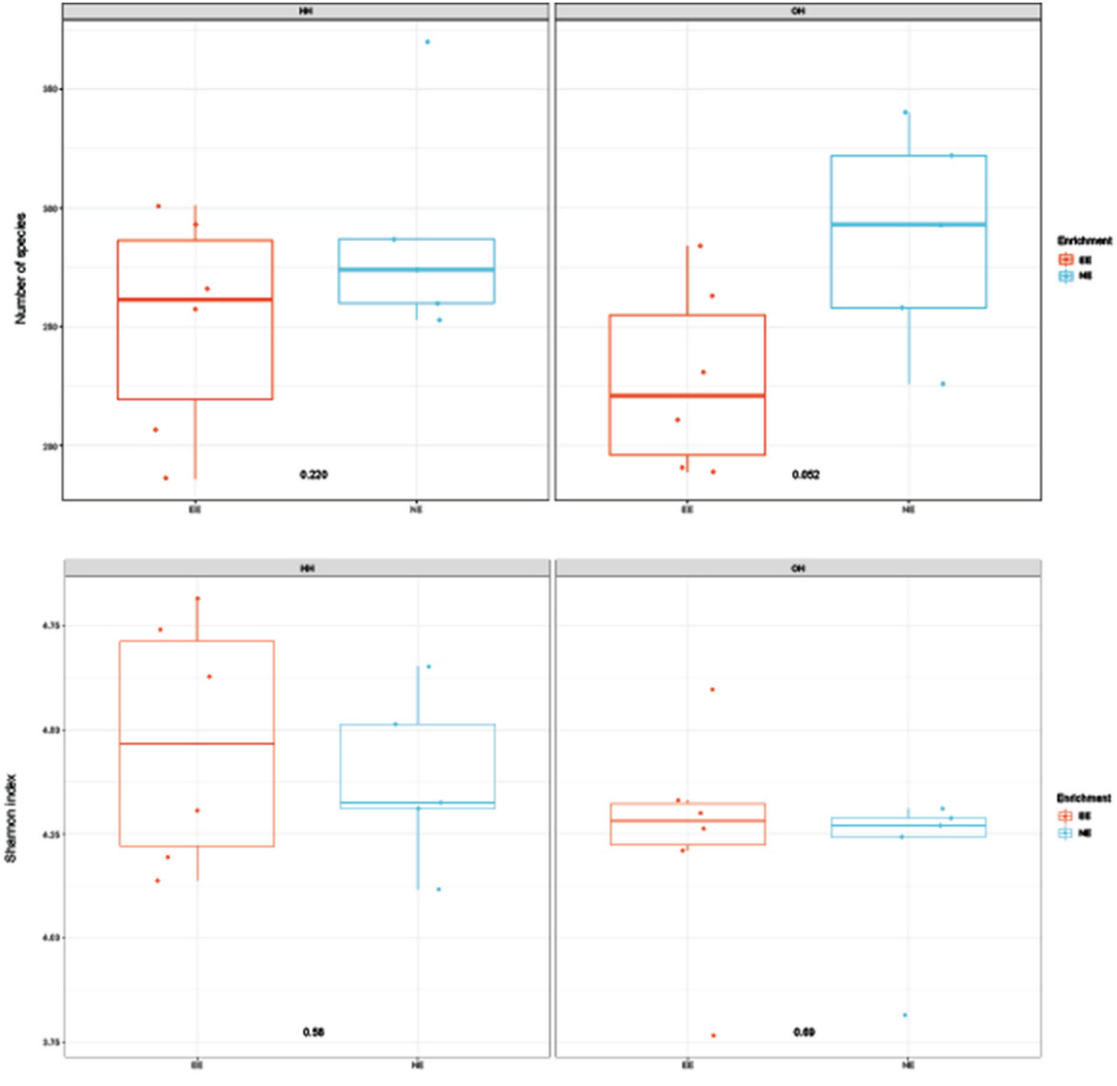
Diversity of microbiota in fecal samples collected on day 14 of age; observed richness (top boxplot) and Shannon index (bottom boxplot). *P*-values of comparison between pens with a dark brooder (EE) and without a brooder (NE) within the hatchery-hatched (HH) resp. on-farm hatched treatments (OH) are indicated in the boxplots. Each point represents a pen (pooled sample of six chickens per pen).

There was no significant interaction between hatching conditions and enrichment for the total number of vocalizations or the latency to the first vocalization. HH chickens vocalized more in the novel environment than OH chickens (*P* <0.05) (Supplementary Figure 1). A significant interaction between enrichment^*^age^*^sex was found (*P* <0.05) (Figure 2). On day 1, NE males vocalized less than NE females, but males and females did not differ in the EE treatment. EE had overall more vocalizations than NE on day 1. On day 11, in the NE treatment, males vocalized less than females, whereas it was the opposite for EE. Overall, differences between NE and EE were smaller on day 11 than on day1 with the highest number of vocalizations for EE compared to NE. An overall effect of age was found with lesser vocalizations on day 11 compared to day 1 (*P* <0.001) (Figure 1; Supplementary Figure 1). Latency to first vocalization did not differ between the treatments and no two- or three-way interactions were found (data not shown). Escape attempts were not observed at day 0 and only 4 attempts were observed on day 11, thus, these (including the latency to escape) were not analyzed.

#### Contact dermatitis and composite asymmetry

Hatching conditions did not affect the prevalence of footpad lesions and hock burn on day 35, but EE broilers had lower footpad lesion and hock burn scores than NE broilers (Supplementary Figure 2) (footpad lesions: *P* <0.001; hock burn *P* <0.001).

The composite asymmetry score on day 35 was significantly lower for EE than NE broilers (49.84 mm vs. 51.96 mm for EE vs NE respectively; se = 0.88; *P* = 0.02). In addition, females had significantly lower composite asymmetry scores than males (19.77 mm vs. 52.02 mm for females vs. males respectively; se = 0.54; *P* <0.001). The hatching system did not affect composite asymmetry scores (data not shown).

### Microbiome composition

#### Pooled fecal samples

**Figure 3:**
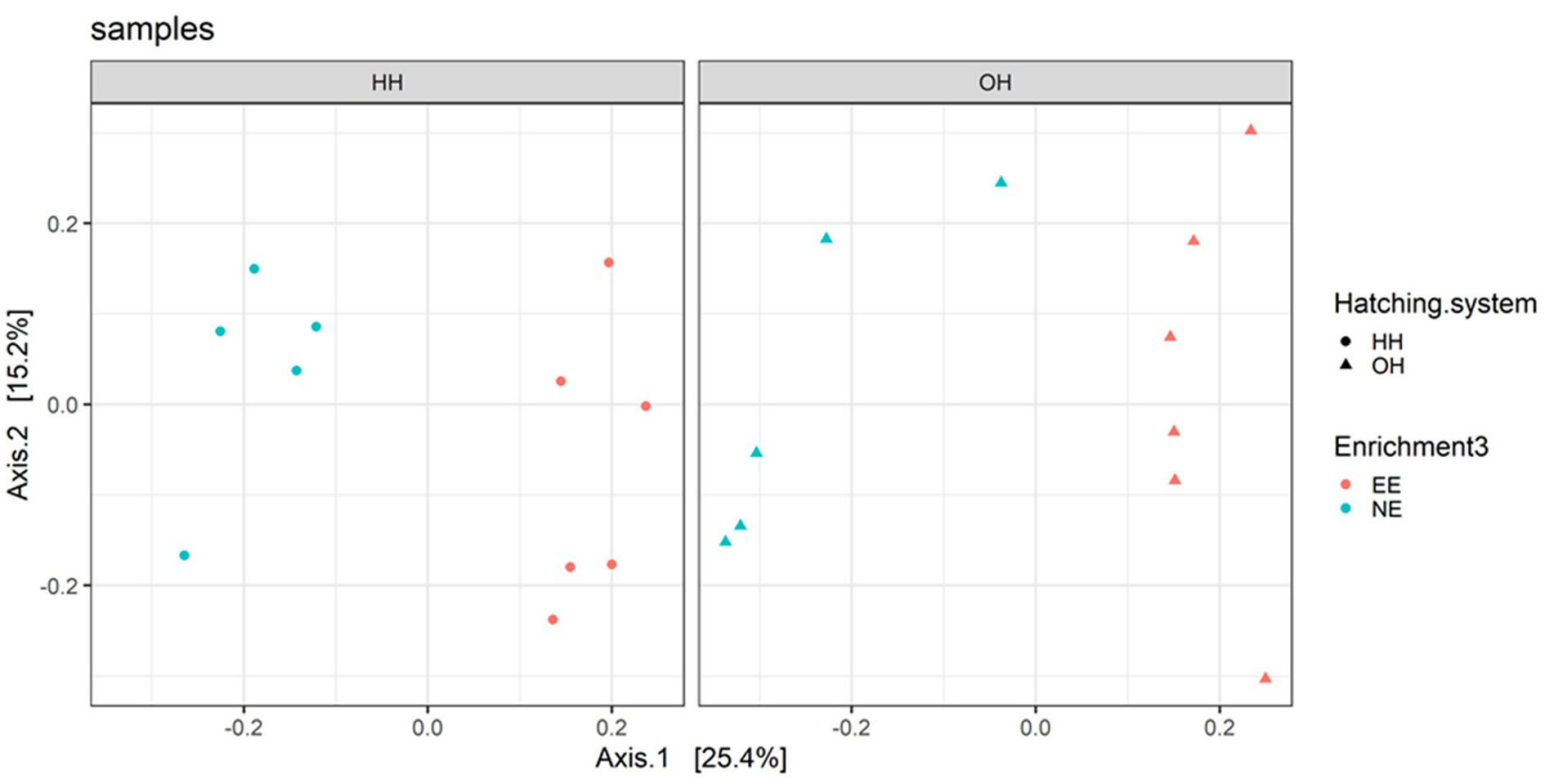
Principal coordinate analysis (family level) of fecal microbiota on day 14 for pens with a dark brooder (EE) and without a brooder (NE) within the hatchery-hatched (HH) respectively, on-farm hatched treatments (OH). Each point represents a pen (pooled sample of six chickens per pen). For significant treatment differences, see text.

The microbiota diversity in fecal samples collected on day 14 was based on the genus/species level data. Within OH, NE showed more species in pooled fecal samples on day 14 compared to EE (Observed richness, *P* = 0.052), but no significant effects were found for the Shannon index (Figure 2). Furthermore, NE did not differ from EE within HH for observed richness and Shannon index. To investigate the microbiota composition as a whole, principal coordinate analysis of the (approximate) family level was performed, which showed a clear separation of EE vs. NE within HH and OH, respectively (Figure 3). Further testing showed that EE differed from NE within HH and OH, that HH-EE differed from OH-NE, and HH-NE from OH-EE (*P* = 0.006 for all comparisons), but HH-EE vs. OH-EE (*P* = 0.357) and HH-NE vs. OH-NE (*P* = 0.460) were not significantly different. No treatment differences were found for the variance (data not shown). When testing for specific bacterial genera between the groups NE vs. EE within HH or OH, significant differences were observed (Table 5). HH broilers with dark brooders (EE) had a higher relative abundance of *Lactobacillus*, but a lower relative abundance of *Corynebacterium, Escherichia/Shigella*, and *Clostriciales_vadinBB60* group than HH-NE. OH broilers with dark brooders (EE) also had a higher relative abundance of *Lactobacillus*, and in addition to *Lachnospiraceae*, and a lower relative abundance of *Staphylococcus, Brachybacterium*, and *Enterococcus* than OH-NE.

**Table 5:** Treatment differences in the relative abundance of the genus-level microbial groups in pooled fecal samples collected on day 14, indicated the significance levels for both without (*P*-value) and with multiple testing (FDR), taking into account.

**Genus**		**Average relative contribution EE (%)**	**Average relative contribution NE (%)**	***P*-value**	**FDR**
**EE vs. NE within HH**					
*Lactobacillus*		20.54	2.97	0.003	0.028
*Corynebacterium_1*		0.69	6.96	0.003	0.028
*Escherichia/Shigella*		5.05	10.47	0.003	0.028
*Clostridiales_vadinBB60_group*		0.43	1.48	0.003	0.028
**EE vs. NE within OH**					
*Lactobacillus*		19.24	2.49	0.003	0.037
*Staphylococcus*		0.49	4.76	0.003	0.037
*Brachybacterium*		0.03	0.86	0.003	0.037
*Enterococcus*		1.39	7.69	0.010	0.085
*Lachnospiraceae*		0.19	0.12	0.013	0.091

EE, dark brooder present until day 14; NE, no dark brooder; HH, hatchery hatching; OH, on-farm hatching.

#### Individual samples of jejunal content

**Figure 4:**
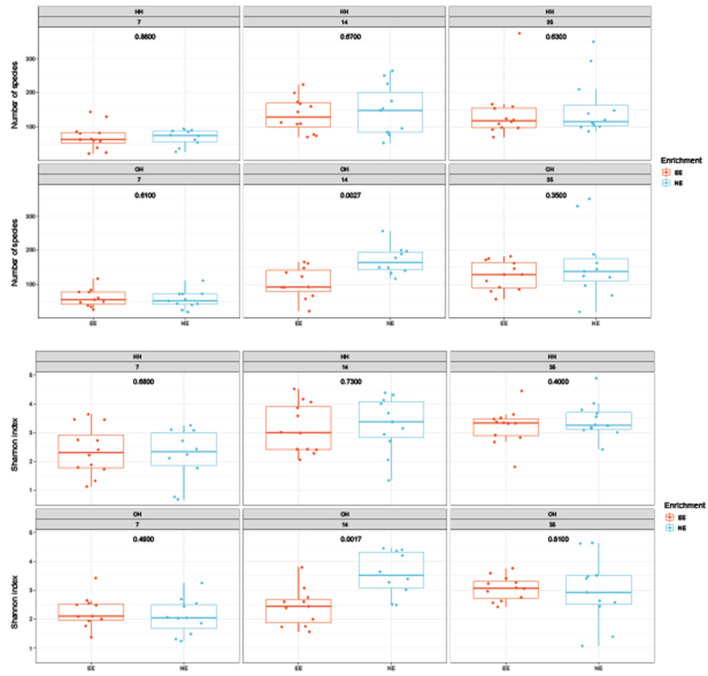
Diversity of microbiota in jejunal samples on days 7, 14, and 35 of age; observed richness (top boxplot) and the Shannon index (bottom boxplot). *P*-values of comparison between pens with a dark brooder (EE) and without a brooder (NE) within the hatchery-hatched (HH) respectively, on-farm hatched treatments (OH) for each age are indicated in the boxplots. Each point represents a chicken.

**Figure 5:**
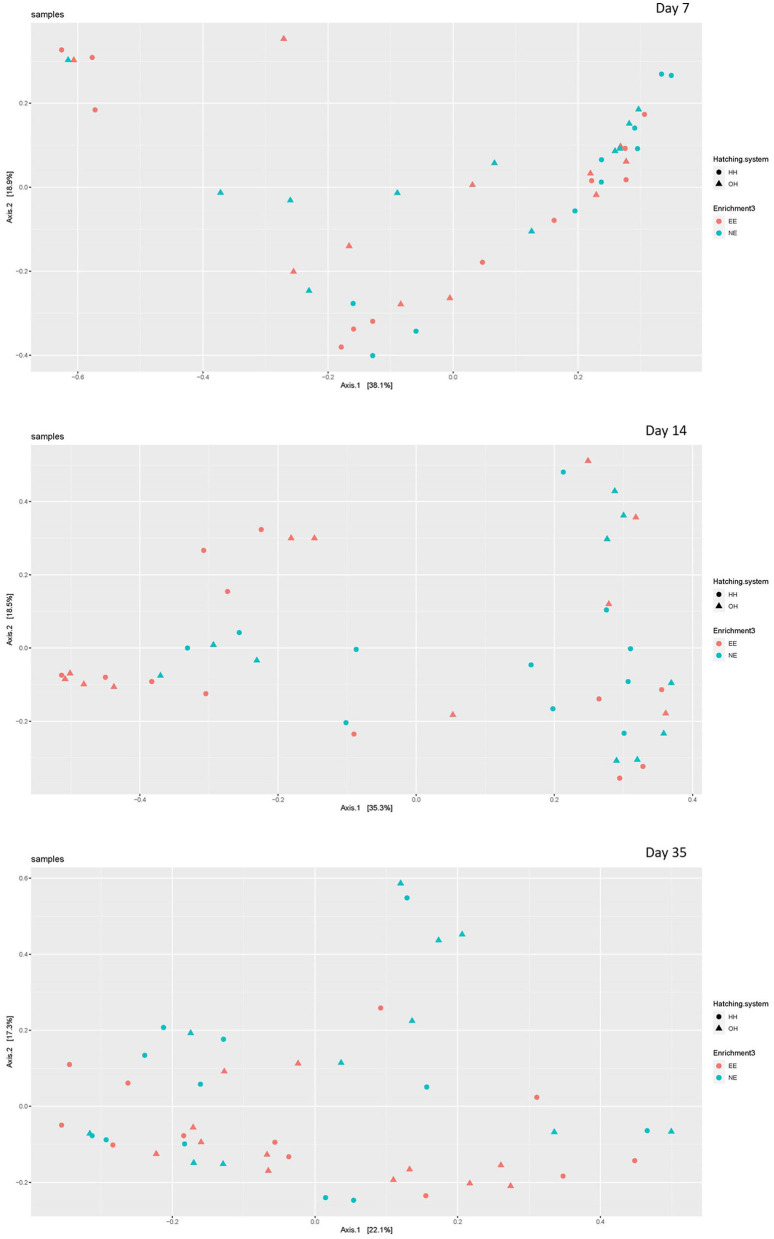
Principal coordinate analysis (family level) of jejunal microbiota on day 7 (upper plot), day 14 (middle plot), and day 35 (bottom plot) for the different treatments [with a dark brooder (EE) and without a brooder (NE), and hatchery-hatched (HH) respectively, on-farm hatched (OH)]. Each point represents a chicken.

The microbial diversity in jejunal samples showed a significantly higher diversity for NE vs. EE within OH on day 14 (Observed richness: *P* = 0.003; Shannon index: *P* = 0.002), but not on days 7 and 35 (Figure 4). In addition, no differences were found for NE vs. EE within HH for both observed richness and the Shannon index. Principal coordinate analysis at the family level was performed for each age (Figure 5). Further testing per time point for all treatment combinations only showed a trend for EE vs. NE within OH on day 35 (corrected *P* = 0.069). The variances between the different groups were not significantly different within a time-point (data not shown). When testing for specific bacterial genera between NE vs. EE within HH or OH per age, no significant differences were observed (data not shown).

## Discussion

In the present study, we determined whether early rearing conditions in broiler chickens not only affected behavior and welfare but also the diversity and composition of the gut microbiome, and possible associations between these. The early rearing environment was modified by different hatching conditions (on-farm hatching vs. traditional hatching at the hatchery) and the presence or absence of a dark brooder until day 14 of age. The hatching environment resulted in few changes in behavior but not in the jejunal and fecal microbiome composition or diversity, whereas the dark brooder treatment showed both short and more long-term effects on behavior, welfare, jejunal, and fecal microbiome composition and diversity. As only a few interaction effects between hatching environment and enrichment were found, these early rearing factors will be discussed separately.

### Effects of a dark brooder in early rearing

A dark brooder resembles the broody hen and provides young chickens with a warm and dark resting place. Providing a brooder in the first 2 weeks of life of laying hens has been shown to increase the bout duration of active and inactive behavior to synchronize behavior (63), reduce fearfulness (53), reduce the risk for injurious pecking (52, 53, 60, 64), and have positive effects on egg production (60) as compared to rearing hens without a brooder. As far as we know, only one study described the use of dark brooders in broiler chickens; in contrast to the laying hen studies, in this particular study, no effects of a dark brooder on behavior or fearfulness were found (65). In the present study, the brooder was well used by the chickens and did have some long-term effects on home pen behavior. On day 33, in the absence of the brooder, fewer EE chickens were sitting and showing comfort behavior, and more EE chickens were eating and pecking at the litter while sitting as compared to NE chickens. Although less sitting, comfort behavior, and eating on days 6 and 13 could be explained by the fact that chickens rested in the brooder, the brooder treatment thus also affected the behavior after it was removed from the pen. Also Riber and Guzman (53) observed less comfort behavior in laying hen chickens provided with a brooder, although they observed more comfort behavior in these chickens on day 42 when the brooder was removed. They suggested that part of the comfort behavior, especially when performed after resting bouts, was performed under the brooder. The lower body weight of EE chickens in the current experiment on day 12 was in line with the observation that fewer EE chickens were eating than NE chickens, but this was compensated thereafter as on day 33 no difference in body weight was observed between the treatments and EE chickens were eating more at an older age. Also, Riber and Guzman (53) observed less eating in laying hen chickens during the first days of age when reared with a dark brooder. They further observed more resting in chickens provided with a dark brooder, supposing that this was the most predominant behavior when being under the brooder. If we assume, for the EE treatment, that both the chickens under the brooder and the chickens sitting idle perform resting behavior, indeed EE chickens rested more on day 6. On day 13 the proportion of resting chickens is more or less equal for EE and NE, while on day 33, NEs were observed to rest more than EE. Thus, our results indicate that the dark brooder can have both a short and long-term effect on broiler chicken home pen behavior, being in line with earlier studies in laying hens.

The frequency of vocalizations in the novel environment was significantly higher in EE than in NE chickens, especially on day 1. Novel environment tests are commonly applied to measure fearfulness in poultry, which is a negative emotional state and thus an important aspect of welfare [e.g., (25)]. During novel environment tests, a high frequency of vocalizations is a behavioral response to regain social contact, whereas a low frequency of vocalizations can be a fearful behavioral response to avoid a predatory threat (i.e., freezing response). The frequency of vocalizations in novel environment tests tends to decline as birds age, probably suggesting an adaptive response to potential predators or threatening scenarios (66, 67). This is confirmed by the lower frequency of vocalizations on day 11 as compared to day 1. Furthermore, it has been suggested that at a very young age, vocalizations are mainly performed to seek social contact (68). Thus, our results indicate a higher social reinstatement in EE than in NE chickens rather than fear of a predator (68). Interestingly, this response is already present on day 1 when the chickens had a relatively short-term experience with the dark brooder. A possible explanation for the difference in response between NE and EE chickens might be the suggestion that dark brooders stimulate sociality in chickens, explaining the higher synchronization of behavior in chickens with a dark brooder (63), which may be regarded as positive because it has been associated with a reduced risk to develop feather pecking and cannibalism in laying hens (52, 53, 60, 64). As in the current experiment, we did not assess fearfulness after day 11, and it is unclear whether EE chickens were indeed less fearful at a later age than NE chickens, as was found in laying hen studies (53).

Fluctuating asymmetry reflects the ability of the chicken to cope with the challenges experienced during rearing and is therefore an indicator of poultry welfare (69, 70). We observed that chickens reared with a dark brooder were more symmetric at slaughter age, which thus indicates that they perceived less stress in the preceding period. Also, better footpad dermatitis and hock burn scores were observed in EE as compared to NE chickens. It is unclear how these effects of a dark brooder on footpad lesion and hock burn prevalence can be explained. Possibly, the difference between NE and EE chickens is related to the growth pattern, as on day 12 NE chickens were heavier than EE chickens, which might have affected the deterioration of the litter quality, which is a major factor related to the prevalence of contact dermatitis (71). It also might be related to the fact that chickens used the brooder for resting in the first weeks, which could have caused the litter quality in the rest of the pen to remain good. Finally, NE chickens were sitting more than EE chickens, which could have had a negative effect on litter quality, and increased the contact time of feet and hocks with the litter, increasing the risk for contact dermatitis.

Rearing with a dark brooder significantly reduced the diversity of the fecal and jejunal microbiome on day 14, and a tendency was found for jejunal samples collected on day 35. This effect was most clear for EE versus NE within OH, and not as strong for EE versus NE within HH. The less diverse microbiome in EE chickens might have been related to the clustering of chickens under the brooder, the decreased feed intake, and the increased resting and reduced comfort behavior when the brooder was present, which could have resulted in a less diverse microbiome as there was less contact with the environment such as litter or feathers. The diet of all treatments was identical so we do not expect that diet itself influenced the diversity, although EE chickens showed less eating behavior. Behavior and health aspects differed between the EE and NE groups. Studies in different species, including humans, showed that a higher microbial diversity might be associated with better wellbeing (72), and that some specific genera in the gut microbiome have an effect on behavior. *Lactobacillus* and *Lachnospiraceae* species are associated with less depression and better health in a human cohort and mice (73) and were more abundant in fecal samples of EE compared to NE. Whether these genera were also associated with better welfare and health in this study is unknown, only that these differed between the treatment groups. Our results were observed in fecal samples and it is not clear whether these genera will also have a different relative abundance in the intestine and how these interact with behavior (74). In other studies, fecal samples were used to investigate depression-like behavior in rats as a proxy of cecal microbiota (75). In our study, we observed differences in behavior but due to the fact that we studied behavior at the pen level, associations of individual behavior with individual differences in bacterial genera could not be analyzed. On day 33 effects of EE on behavior were absent or opposite to the earlier ages, and this could explain why on day 35 there was only a trend for a difference between the jejunal microbiome diversity in the EE and NE. Moreover, EE chickens were showing more eating and pecking at the litter while sitting on day 33 compared to NE, which could have attributed to the increase in jejunal microbial diversity. It can only be speculated why the difference in microbiome diversity and composition between EE and NE seemed to be larger within the on-farm hatched chickens. As chickens can already acquire microorganisms in the hatchery and during transport (33), this could have led to the colonization of the gut in the HH chickens before they could access the dark brooder and thus result in a reduced effect of the brooder on microbial diversity and composition during early rearing. On the other hand, the effects of the dark brooder on microbial diversity and composition were observed from day 14 onward, 2 weeks after the hatching treatment. Early feeding or probiotic treatments are known to affect early colonization and the microbiome later in life, and this influences intestinal development (76, 77). Whether environmental enrichment, i.e., the dark brooder, which is not a nutritional intervention, gives an additional effect to this early colonization resulting in a difference in intestinal development later in life, needs to be further investigated.

The results of the present study support suggestions from earlier studies indicating that the rearing environment alone can affect gut microbiome composition and diversity (21–23), even when all other conditions such as diet are identical. While earlier studies showed that barren housing conditions in laying hens resulted in a less diverse gut microbiome than more enriched housing, and associated the lower gut microbial diversity with reduced normal behavior and impaired welfare (21–23), our results suggest the opposite, i.e., EE broilers having a lower diversity and better welfare than NE broilers. However, the dark brooder in the present experiment was only applied for the first 2 weeks, whereas the hens in previous studies were housed in the respective barren and enriched housing conditions for a (much) longer period. It could be that the effects of the dark brooder on gut microbiome composition and diversity were transient, as the microbiome has not yet become stable, e.g., in laying hens, a stable microbiome composition was observed from day 50 onward (78). In addition, we cannot say yet whether the early rearing environment affects the microbiome, behavior, and welfare, or whether the gut–brain axis plays a role in the effects on the behavior *via* the changes in microbial composition as a result of the dark brooder treatment. For example, it remains to be further determined whether the differences in sociality on day 11 in the EE vs. the NE groups could be linked to an altered microbiome composition, as these effects on sociality were already present on day 1, while the gut microbiome was only different from day 14 onward, and sociality was not measured after day 11. In studies where the microbiome was altered due to antibiotics or where germ-free mice were used, differences in social interactions were found [e.g., (79, 80)]. However, in our study, the microbiome was not altered as an intervention, but the effect of the dark brooder was that the EE group showed more social reinstatement. In chimpanzees, the microbiome was more uniform between chimpanzees with more social interaction, although in that study diet most probably interacted with the microbiome results (81). It remains to be further determined whether there is a relationship between microbiome composition and social behavior in EE chickens, or whether the observed effects on sociality are a result of early programming of behavior due to the dark brooder in the pen.

### Effect of hatching conditions

On-farm hatching in the present experiment only resulted in significantly higher body weight at day 0 but not thereafter, which is in contrast with previous studies indicating a more long-term effect of on-farm hatching on body weight development in fast-growing broiler chickens (38–41). Lower body weight in HH as compared to OH chickens is likely caused by the delay in access to feed and water (82). Although studies showed that the duration of the effect of the first feeding moment on body weight may vary, due to variation in post-hatch feed deprivation time and the ability of delayed-fed chickens to show compensatory growth (82, 83), on-farm hatched chickens usually show a higher body weight at least during the first weeks of life (38–41). It is unclear why we observed such short-term effects of hatching conditions on body weight development in the present experiment. Possibly, the HH chickens hatched relatively late, which, in combination with a relatively short transport time (1 h), resulted in a relatively short post-hatch feed deprivation. Unfortunately, we have no information on the hatching moment of the HH chickens. In addition, the first OH chickens started to hatch on day 20, which indicates that the difference in the first feeding moment between the treatment groups might have been relatively small, which could explain the absence of a long-lasting effect on body weight (82). In commercial practice, transport times might be several h, resulting in more long-term negative effects of delay in first feeding on body weight (41) than in the current experiment.

A higher frequency of vocalizations was observed for HH as compared to OH chickens in the novel environment. As explained above, it has been suggested that at a very young age, vocalizations are mainly performed to seek social contact (68). Thus, on day 1, the higher frequency of vocalizations in HH chickens may indicate a higher motivation for social reinstatement in HH as compared to OH chickens, which is in line with a previous study comparing on-farm with hatchery hatched broiler chickens (44), but was not verified in a later study by the same group (45). The difference in vocalizations between HH and OH was smaller, but still present, on day 11. Regarding home pen behavior, HH chickens showed more locomotion than OH chickens, which is in line with other studies (44, 45), but other behaviors were not affected by hatchery treatment. Furthermore, no significant effects of hatching conditions were observed on the prevalence of footpad dermatitis and hock burn, in contrast to results of previous studies where on-farm hatching was shown to reduce footpad dermatitis (39, 41, 45). The composite asymmetry score, an indicator of stress during development (69, 70), was also not affected by the hatching treatment. Taken together, on-farm hatching as practiced in the present experiment had only minor effects on behavior and welfare indicators.

Since after the first ingestion of feed there is a significant increase in microorganisms in the chicken intestine (4), the moment of first feeding could affect the intestinal and fecal microbiome composition. On-farm hatched chickens could immediately access feed and water post-hatch and were therefore expected to have faster colonization of the gut as compared to hatchery-hatched chickens. Walstra (24) observed that early-fed laying hen chickens differed in cecal microbiome composition until 62 days of age from hens that were post-hatch feed deprived for 72 h, although the ileal microbiome composition only differed on day 3 of age but not at later ages. Simon (84) found that broiler and laying hen chickens fed immediately post-hatch differed in ileal microbiome composition from chickens that were post-hatch feed deprived for 72 h at both 3 and 9 days of age, but did not find significant differences from day 21 of age onward. In the present study, we did not observe significant differences in microbiome diversity and composition between HH and OH at all ages. Possibly, only a short-lasting delay in colonization of the gut between HH and OH was present, as both groups were housed in an identical environment from day 0 onward and received an identical diet, which resulted in similar colonization, which was only delayed in the HH group. Thus, the first moment of analysis of the microbiome (day 7 of age) might have been too late to observe any differences between the hatching conditions. It cannot be excluded that other environmental factors also played a role in the microbial colonization of the gut in addition to the diet; HH and OH broilers were from the same parent stock and housed in the identical environment from day 0 onward. The different environment between embryonic day 18 and day 0 of age and disinfection of the HH chickens in the hatchery seemed to play a minor role in the microbial colonization of the gut in the present study.

## Conclusion

In the present study, we showed that rearing broiler chickens with a dark brooder from days 0–14 affects sociality, behavior, welfare indicators, and the gut microbiome, as compared to rearing broilers without a brooder, and that some of these effects last until slaughter age (d35). However, whether there is just an association between behavior, welfare, and gut microbiome diversity or composition, or whether the microbiome modulates the behavior or vice versa needs to be further studied. Contrary to the expectations, hatching conditions affected sociality and had a minor effect on home pen behavior, but did not change the gut microbiome composition. The relatively short transport duration and thus likely the relatively small difference in timing of first feeding between HH and OH may have contributed to the lack of effects of hatching conditions on the gut microbiome composition.

## Data availability statement

The datasets presented in this study can be found in online repositories. The names of the repository/repositories and accession number(s) can be found below: https://www.ncbi.nlm.nih.gov/, PRJNA854099. All other data can be provided upon request.

## Ethics statement

The animal study was reviewed and approved by Ethical Committee of Wageningen Livestock Research, Wageningen University and Research.

## Author contributions

IJ and JR contributed to the conception and design of the study. IJ, MW, and HG conducted the animal experiment. IJ and DS performed the statistical analyses. IJ wrote the first draft of the manuscript. JR and DS wrote sections of the manuscript. IJ, JR, and DS contributed to the manuscript revision. All authors read and approved the submitted version.

## Funding

The present study was funded by the Ministry of Agriculture, Nature and Food Quality within the program KB-34-006-008 Microbiome Program.

## Conflict of interest

The authors declare that the research was conducted in the absence of any commercial or financial relationships that could be construed as a potential conflict of interest.

## Publisher's note

All claims expressed in this article are solely those of the authors and do not necessarily represent those of their affiliated organizations, or those of the publisher, the editors and the reviewers. Any product that may be evaluated in this article, or claim that may be made by its manufacturer, is not guaranteed or endorsed by the publisher.
