# Supplementary material for: Early life environment affects behavior, welfare, gut microbiome composition, and diversity in broiler chickens
Source: Front Vet Sci. 2022 Sep 12;9:977359. doi: 10.3389/fvets.2022.977359 (PMC9534479; doi:10.3389/fvets.2022.977359)
Supplement: Supplementary file 1 [file Image_1.pdf]

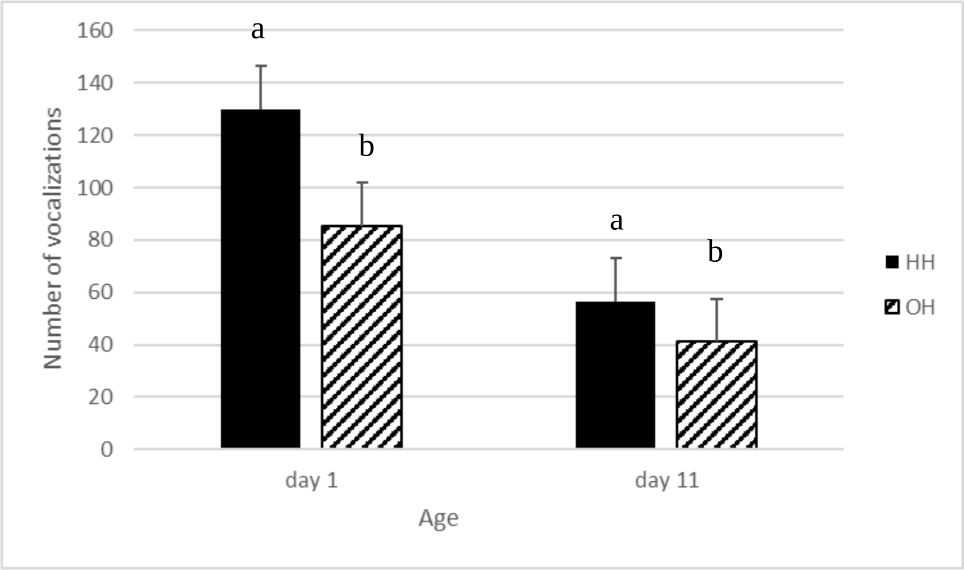

Supplementary Figure 1. Average frequency of vocalizations ± se in the novel environment test at d1 and 11 of age, for chickens that hatched in the hatchery (HH) or on-farm (OH). HH vocalized significantly more than OH ($P<0.05$), indicated by different letters above the bars per age.
