# Supplementary material for: Early life environment affects behavior, welfare, gut microbiome composition, and diversity in broiler chickens
Source: Front Vet Sci. 2022 Sep 12;9:977359. doi: 10.3389/fvets.2022.977359 (PMC9534479; doi:10.3389/fvets.2022.977359)
Supplement: Supplementary file 2 [file Image_2.pdf]

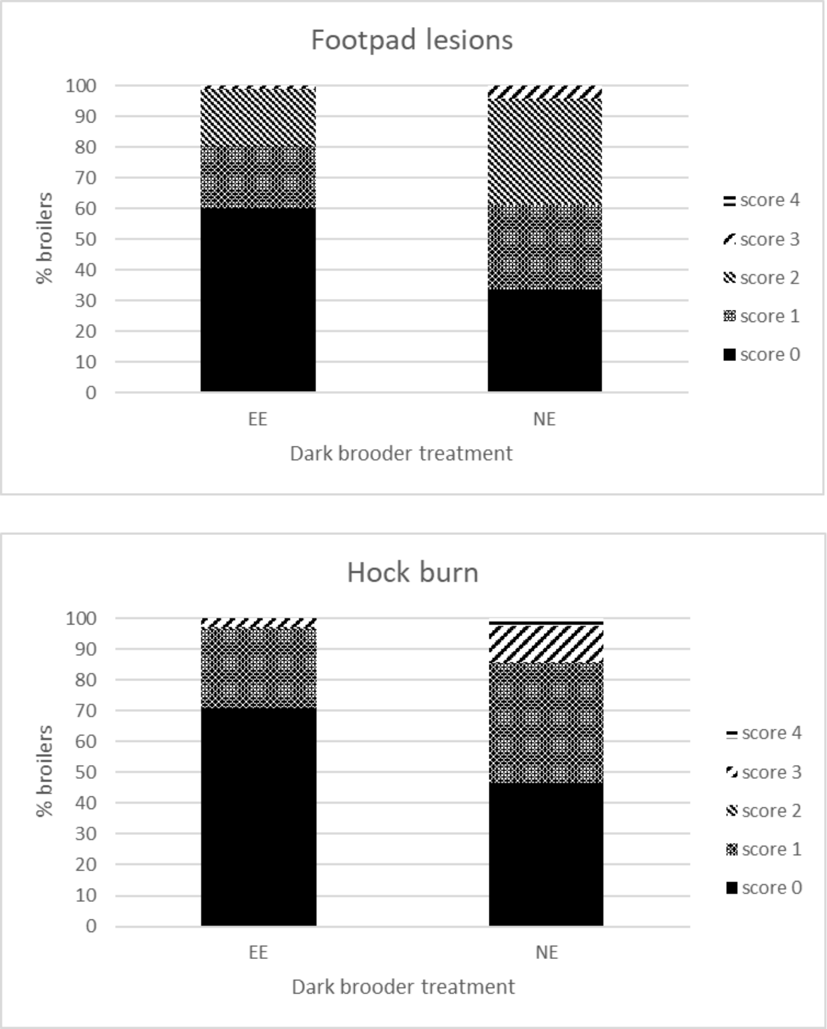

Supplementary Figure 2. Distribution of footpad lesion scores and hock burn scores for NE (no brooder) and EE (dark brooder until d14). Higher scores indicating more severe lesions. NE had significantly higher (worse) footpad lesion and hock burn scores than EE (P<0.001).
